# Regulation of RNA Polymerase II Termination by Phosphorylation of Gdown1*

**DOI:** 10.1074/jbc.M113.537662

**Published:** 2014-03-14

**Authors:** Jiannan Guo, Michael E. Turek, David H. Price

**Affiliations:** From the Department of Biochemistry, University of Iowa, Iowa City, Iowa 52242

**Keywords:** Mitosis, Phosphorylation, RNA Polymerase II, Transcription Elongation Factors, Transcription Termination, Gdown1, TFIIF, TTF2

## Abstract

Gdown1 is a substoichiometric subunit of RNA polymerase II (Pol II) that has been recently demonstrated to be involved in stabilizing promoter-proximal paused Pol II. It was shown to inhibit termination of Pol II by transcription termination factor 2 (TTF2) as well as block elongation stimulation by transcription factor IIF (TFIIF). Here, using *in vitro* transcription assays, we identified two functional domains in Gdown1. Although both are required to maintain a tight association with Pol II, the N- and C-terminal domains are responsible for blocking TTF2 and TFIIF, respectively. A highly conserved LPDKG motif found in the N-terminal domain of Gdown1 is also highly conserved in TTF2. Deletion of this motif eliminated the TTF2 inhibitory activity of Gdown1. We identified a phosphorylated form of Gdown1 with altered mobility in SDS-PAGE that appears during mitosis. A kinase in HeLa nuclear extract that caused the shift was partially purified. *In vitro*, Gdown1 phosphorylated by this kinase demonstrated reduced activity in blocking both TTF2 and TFIIF because of its reduced affinity for Pol II. Mass spectrometry identified Ser-270 as the site of this phosphorylation. An S270A mutation was not phosphorylated by the partially purified kinase, and an S270E mutation partially mimicked the properties of phospho-Gdown1. Gdown1 Ser-270 phosphorylation occurs predominately during mitosis, and we suggest that this would enable TTF2 to terminate all Pol II even if it is associated with Gdown1.

## Teaser

**Background:** Gdown1 is a substoichiometric subunit of RNA polymerase II that blocks termination by TTF2 and the function of TFIIF during initiation and elongation.

**Results:** Domains of Gdown1 responsible for inhibiting TTF2 and TFIIF as well as a controlling phosphorylation site are identified.

**Conclusion:** Gdown1 can be regulated by phosphorylation of Ser-270.

**Significance:** Our results reveal a mechanism that allows termination of all polymerases during mitosis.

## Introduction

Regulation of Pol II^2^ transcription elongation is crucial in regulating gene expression in eukaryotes (1). In human, a prominent feature of Pol II transcription is polymerase pausing at promoter-proximal locations after initiation and promoter clearance. The pause is detected shortly after Pol II enters elongation, approximately 50 bp downstream from the transcription start site (2). This pause can be alleviated by the kinase activity of P-TEFb, which triggers the transition into productive elongation (3, 4). During productive elongation, Pol II elongates the nascent transcript at the rate of 2–6 kb/min (5) and carries out co-transcriptional RNA processing events to generate mature mRNAs (6). Significantly, promoter-proximal paused Pol II accounts for the majority of engaged Pol II in human cells and can be seen as a reservoir that can respond to activation signals (1).

Factors involved in generating and maintaining promoter-proximal paused Pol II include the 5,6-dichloro-1-β-d-ribofuranosylbenzimidazole (DRB) sensitivity-inducing factor DSIF, the negative elongation factor NELF, Gdown1, and the Gdown1 negative accessory factor GNAF. DSIF is required for the P-TEFb inhibitor (DRB) to convey a negative effect on elongation (7). NELF cooperates with DSIF to exhibit a strong negative effect on elongation (8–10). Upon the action of P-TEFb, NELF is released from the elongation complex (11, 12) whereas DSIF is phosphorylated and remains associated with Pol II (13, 14). Recently, Gdown1, a substoichiometric subunit of Pol II (15) along with an unidentified accessory factor GNAF (16), has been shown to stabilize promoter-proximal paused Pol II. Gdown1 inhibits elongation stimulation by TFIIF and termination by TTF2 (16). Cross-linking assays and electron microscopy showed that Gdown1 directly interacts with the Rpb1 and Rpb5 subunits of Pol II in a way that would sterically exclude TFIIF (17, 18). Gdown1 associates with Pol II stably and outcompetes TFIIF and TTF2. When Gdown1 is bound to an isolated early elongation complex (EEC), the resulting EEC(G) is resistant to high salt wash up to 1.6 m KCl (16). ChIP-Seq data showed that Gdown1 can be found over gene bodies of many highly transcribed genes, indicating that Gdown1 can be present in the elongation complex (16, 17). We hypothesized previously that there must be a mechanism to regulate Gdown1 function because stably bound Pol II(G) poses a problem during mitosis when all polymerases must be terminated by the action of TTF2 (16, 19).

Here we dissected the functional domains of Gdown1 and found that the N-terminal domain (NTD) of Gdown1 is required to block TTF2 and the C-terminal domain (CTD) is responsible for blocking TFIIF. We also identified a phosphorylation event that reduces the affinity of Gdown1 with Pol II, thereby allowing TFIIF and TTF2 to function on elongation complexes. These results provide mechanistic insights toward the function and regulation of Gdown1 during mitosis and perhaps during mRNA production.

## EXPERIMENTAL PROCEDURES

### 

#### 

##### Gdown1 Constructs and Protein Purifications

Full-length human Gdown1 in the pET100 directional TOPO vector (Invitrogen) was described previously (16). Gdown1 truncation mutants containing amino acids 1–89 (NTD) and 197–368 (CTD) were generated by PCR and cloned into the same pET100 vector. The NTD clone generated also included the sequence ERSGC following amino acid 89 of Gdown1. Site-specific mutations in Gdown1 were generated using QuikChange II site-directed mutagenesis (Stratagene) following the manufacturer's recommendations. For the ΔLPDKG deletion mutant, the sequence encoding LPDKG was deleted from full-length Gdown1. For the S270A and S270E mutations, the Ser-270 codon (TCT) was changed to alanine (GCC) and glutamic acid (GAA), respectively. The proteins were expressed in BL21 star (DE3) and purified by nickel-nitrilotriacetic acid followed by Mono Q (except the NTD was purified on Mono S) using methods described earlier (16).

##### In Vitro Transcription Assays

Generation of EECs was described previously (20, 21). In brief, an immobilized CMV promoter containing template was incubated with HeLa nuclear extract (HNE) (22), and initiation was accomplished with a 30-s pulse with 500 μm A/U/GTP spiked with [α-^32^P]CTP. After stopping the reaction with EDTA the elongation complexes were washed with 1.6 m salt to remove associated factors. For add-backs, purified wild-type or truncated/mutated versions of Gdown1, human TFIIF (23), and human TTF2 (19) were added to the reactions and incubated for 5 min at room temperature. The reactions were then optionally washed with the indicated salt solutions before being chased with 500 μm cold NTPs for the indicated times. Labeled transcripts were analyzed on denaturing RNA gels (6 m urea) followed by phosphorimaging.

Quantification of the effects of TFIIF and TTF2 was accomplished by quantifying runoff for the effect of TFIIF and quantifying the percentage of the total transcripts present in a region of the gel containing most of the terminated transcripts for TTF2. This was carried out using version 3 of the MultiGauge software (FujiFilm) supplied with the FLA7000 Phosphorimager (GE Healthcare). Although analyzing the effect of TTF2 was complicated by the presence of a small amount of nonterminated transcripts in the region in which terminated transcripts appeared, the relative percentage in this region between lanes is a good estimate of the relative termination activity between lanes.

##### EC-EMSA

Early elongation complexes were generated with the CMV promoter template with a runoff point at +183 as described previously (24). 1 pmol of Gdown1 (WT, mutant, or phospho-G) was incubated with the complexes for 10 min before being subjected to the indicated washes. The elongation complexes carrying nascent transcripts were digested off the beads with 10 units of SacI-HF per reaction in low salt wash (LSW) buffer supplemented with 10 mm MgCl_2_. The complexes in solution were run on 4% acrylamide gel in 0.5 × Tris/glycine buffer for 2.5 h at 6 W and dried before phosphorimaging.

##### HeLa Cell Mitotic Shake Off

HeLa cells were grown in DMEM supplemented with 10% FBS. Medium was replaced several hours before the first shake off. Vigorous tapping on the sides of a T-150 flask released mitotic cells that were then spun down (1200 rpm in Beckman J-6) before being washed in and resuspended with ice-cold PBS. Cells remaining on the plate were scraped in medium, washed, and resuspended with PBS. Mitotic and interphase cells were lysed with SDS containing protein sample buffer, and aliquots were analyzed by SDS-PAGE (9% acrylamide).

##### Fractionation of HNE and Kinase Assays

0.5 ml of HNE was fractionated on Mono S with a gradient of 100 mm to 1 m KCl in HGKEDP (25 mm HEPES, 15% glycerol, indicated KCl, 0.1 mm EDTA, 1 mm DTT, and 0.1% of a saturated solution of PMSF in isopropyl alcohol). Each collected fraction was used in the kinase assay to phosphorylate recombinant Gdown1. The fractions able to phosphorylate Gdown1 and change its mobility by SDS-PAGE were pooled and further fractionated on Mono Q with a gradient of 100 mm to 1 m KCl. In the kinase assays, each reaction included the indicated amounts of Gdown1 and 4 μl of each fraction in 20 mm HEPES (pH 7.6), 60 mm KCl, 5 mm MgCl_2_, 1 mm ATP (spiked with [γ-^32^P]ATP) and was incubated at 37 °C for 1 h. The reactions were then analyzed by SDS-PAGE followed by silver staining and phosphorimaging.

##### Mass Spectrometry Analyses

10 μg of Gdown1 and 100 μl of Mono Q fraction 19 were incubated in 20 mm HEPES (pH 7.6), 50 mm KCl, 5 mm MgCl_2_, and 1 mm ATP at 37 °C for 4 h. The Gdown1 was then purified by nickel-nitrilotriacetic acid before being dialyzed into 50 mm (NH_4_)HCO_3_ and subjected to mass spectrometry using the method described in Ref. 25 to identify sites of phosphorylation.

## RESULTS

### 

#### 

##### Conservation Suggests Functional Domains of Gdown1

**FIGURE 1.**
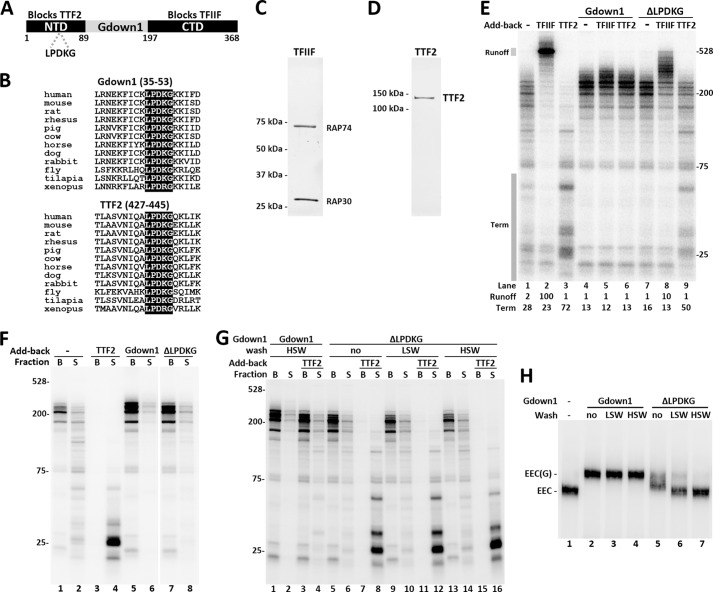
**The LPDKG motif in the Gdown1 NTD is required for TTF2 inhibition and tight association with Pol II.** *A*, diagram of Gdown1 indicating the conserved NTD and CTD with their proposed respective functions. The regions that were cloned to make truncation proteins are *highlighted* and have amino acid numbers marked. Location of the LPDKG motif is indicated. *B*, protein sequence alignments of Gdown1 and TTF2 in example species around the LPDKG motif. *C*, silver staining of purified TFIIF (consisting RAP74 and RAP30 subunits). *D*, silver staining of purified recombinant human TTF2 protein. *E*, *in vitro* transcription reactions comparing the effects of Gdown1 (1 pmol/reaction) and the ΔLPDKG mutant (1 pmol/reaction) on TFIIF (0.1 pmol/reaction) and TTF2 (0.04 pmol/reaction) during elongation. Isolated EECs were incubated with indicated factors before a 7-min chase. RNA was isolated and then analyzed on a denaturing gel. The labeled transcripts shown here were detected by phosphorimaging. Transcript sizes are indicated in nucleotides. Intensity of the runoff region (indicated by a *shaded box*) in each *lane* was quantified and normalized against *lane 2*. The termination region (indicated by a *shaded box*) in each *lane* was quantified and is shown as its percentage of the total lane intensity. *F*, *in vitro* transcription reaction similar to *E*, except that TTF2 was 0.08 pmol/reaction and after stopping elongation. Beads (*B*, engaged complexes) and supernatant (*S*, terminated complexes) were separated and run separately. *G*, isolated EECs were incubated with 1 pmol of Gdown1 or the ΔLPDKG mutant before being subjected to no wash or two rounds or washing with 60 mm KCl (LSW) or 1.6 m KCl (HSW). The resulting complexes were supplemented with TTF2 (0.08 pmol/reaction) as indicated before a 7-min chase. Beads and supernatant were separated after the chase. *H*, EC-EMSA showing Gdown1 associating with EECs. Isolated EECs were incubated with 1 pmol of Gdown1 or the ΔLPDKG mutant before LSW or HSW as indicated. The complexes were digested off the beads by SacI at 37 °C for 15 min and run on a 4% native acrylamide gel for 2.5 h at 6 W. Bands corresponding to EEC or EEC(G) are indicated.

To identify functional domains of Gdown1, we carried out a sequence alignment of Gdown1 proteins from species ranging from insects to primates. Sequence similarities indicated conservation in an NTD and in a CTD as illustrated in Fig. 1*A*. The NTD contains the most highly conserved peptide in Gdown1, LPDKG. The identical sequence is found in all species, except *Xenopus*, which contains a similar peptide LPDRG (Fig. 1*B*, *Gdown1*). Interestingly, an alignment of TTF2 from a wide range of species also shows a highly conserved LPDKG pentapeptide within a well conserved region of TTF2 (Fig. 1*B*, *TTF2*). TTF2 from *Xenopus* had the same LPDRG peptide found in *Xenopus* Gdown1, which suggests co-evolution of the motif. We hypothesize that the NTD of Gdown1 competes with TTF2 for Pol II binding.

##### The NTD of Gdown1 Is Required to Block TTF2 and for Tight Gdown1 Binding to Pol II

We generated a ΔLPDKG mutant with the sequence deleted from otherwise full-length Gdown1 to determine whether the motif played a role in blocking termination by TTF2. The purified recombinant protein was tested using an *in vitro* transcription assay (21). TFIIF (Fig. 1*C*) and TTF2 (Fig. 1*D*) are known to be inhibited by Gdown1 during elongation (16) and, therefore, used here to assay the strength of Gdown1 and its variants. EECs were generated by incubating the immobilized template in HeLa nuclear extract and performing a 30-s pulse with limiting [α-^32^P]CTP. The complexes generated contained transcripts that were shorter than 25 nucleotides. These EECs were washed three times with a buffer containing 1.6 m KCl to remove associated factors. Chasing the EECs with 500 μm NTPs for 7 min produced transcripts up to ∼200 nucleotides in length (Fig. 1*E*, *lane 1*). As expected (16, 21, 26), reactions supplemented with TFIIF showed a dramatic stimulation of elongation (Fig. 1*E*, *lane 2*), whereas addition of TTF2 caused termination of the EECs resulting is a characteristic pattern of shorter transcripts, which have been demonstrated to be terminated transcripts released from the template (16, 19) (Fig. 1*E*, *lane 3*). Note that there is a small amount of TTF2 that remains bound to the template during high salt wash, and this leads to a low level of termination during the chase (compare *lanes 1* and *3*). Addition of Gdown1 eliminated the effect of the contaminating TTF2 resulting in a pattern of slightly longer transcripts compared with Pol II alone and the disappearance of shorter transcripts (Fig. 1*E*, *lane 4*, *Term* region). As expected, Gdown1 blocked the effects of both TFIIF and TTF2 (16) (Fig. 1*E*, *lanes 5* and *6*). As determined by quantifying the amount of runoff, the ΔLPDKG mutant blocked 90% of the TFIIF-dependent runoff (Fig. 1*E*, compare *lanes 2* and *8*). However, the ΔLPDKG mutant only weakly blocked TTF2 as determined by quantifying the level of short transcripts caused by termination. The region quantified was 72% of the total counts with TTF2 alone and 50% with TTF2 in the presence of the ΔLPDKG mutant, which means more than half of the total termination activity remained (Fig. 1*E*, compare *lanes 3* and *9*). Although the ΔLPDKG mutant affected both TFIIF and TTF2, it blocked TFIIF significantly more than it blocked TTF2, suggesting that the NTD is primarily involved in blocking TTF2.

The property of the ΔLPDKG mutant was further characterized by separating the bead-bound elongation complexes and the supernatant containing terminated complexes after the reactions. As shown in Fig. 1*F*, EECs left a moderate trail of termination products during the chase (*lanes 1* and *2*) whereas addition of TTF2 terminated most of the complexes at early stage (*lanes 3* and *4*). Addition of 1 pmol of either the wild-type Gdown1 or the ΔLPDKG mutant significantly increased the proportion of engaged complexes, with ΔLPDKG mutant showing slightly more terminated transcripts because of the low level of TTF2 in the isolated complexes (*lanes 5–8*). To test ΔLPDKG mutant affinity to Pol II, salt washes with 60 mm KCl (LSW) or 1.6 m KCl (HSW) were introduced after incubating the Gdown1 with the EECs and before TTF2 addition and the chase (Fig. 1*G*). As had been demonstrated previously (16), Gdown1 is resistant to HSW, and therefore TTF2 remained mostly inhibited (*lanes 1–4*). However, the ΔLPDKG mutant, regardless of no wash, LSW or HSW, did not show significant inhibition against the strong termination activity from TTF2 (0.08 pmol). This suggests the ΔLPDKG mutant does not associate with Pol II as tightly as wild-type Gdown1.

To confirm the lowered binding of the ΔLPDKG mutant to Pol II we carried out electrophoretic mobility shift assays with isolated elongation complexes (EC-EMSA) (24). As shown previously (16), wild-type Gdown1 bound to Pol II causing a lowered mobility band detected by the labeled RNA in the elongation complex. Also as expected the binding was resistant to LSW or HSW (Fig. 1*H*, *lanes 1–4*). The ΔLPDKG mutant, when in excess (1 pmol), created a more heterogeneous shift with some density in the fully shifted position and most density in a band of intermediate mobility between the elongation complex alone and the Gdown1-bound complex (Fig. 1*H*, *lane 5*). This suggests that the ΔLPDKG mutant binds differently to the elongation complex and may exist primarily in an altered conformation with the CTD bound to Pol II and the NTD only weakly associated with Pol II. After LSW, most of the complexes had the mobility of free elongation complexes, and after HSW, the shifted complexes became undetectable (Fig. 1 1*H*, *lanes 5–7*). From all of these results we conclude that the LPDKG motif is involved in inhibition of TTF2-mediated termination and that the interaction of Gdown1 with Pol II is weakened by the mutation.

##### The CTD of Gdown1 Blocks TFIIF Function

**FIGURE 2.**
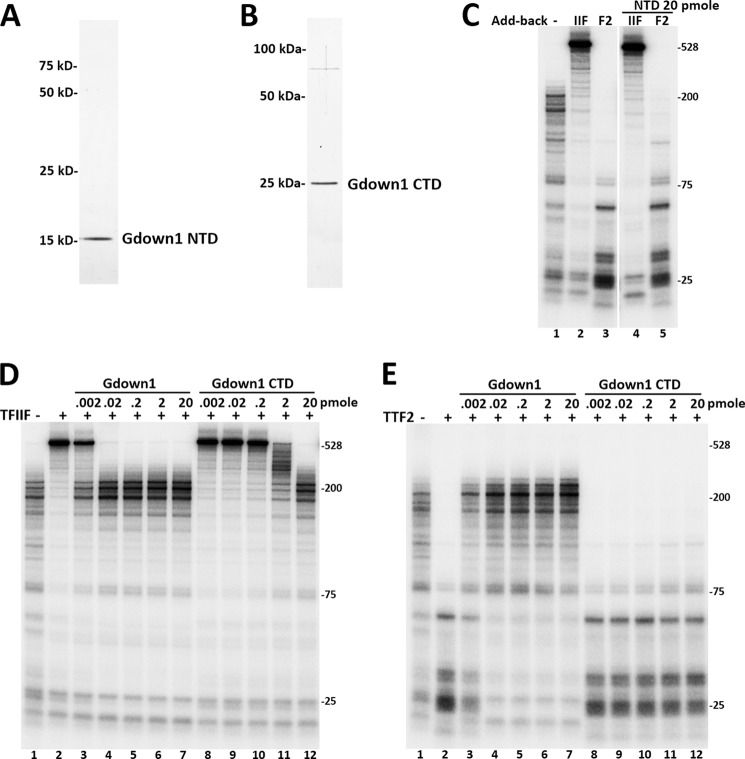
**The Gdown1 CTD blocks TFIIF but not TTF2.** *A*, silver staining of purified recombinant Gdown1 NTD(1–89). *B*, silver staining of purified recombinant Gdown1 CTD(197–368). *C*, indicated amount of Gdown1 NTD incubated with EECs before adding in TFIIF or TTF2 and chased for 7 min. *D* and *E*, indicated amounts of Gdown1 (full-length) or Gdown1 CTD(197–368) incubated with EECs and 0.1 pmol of TFIIF (*D*) or 0.04 pmol of TTF2 (*E*) as indicated before reactions were chased for 7 min. Isolated RNA was separated on a denaturing gel, and the labeled transcripts were detected by phosphorimaging.

To assess the functions of the Gdown1 individual domains, truncation proteins containing the NTD (amino acids 1–89) and CTD (amino acids 197–364) of Gdown1 were cloned, expressed, and purified (Fig. 2, *A* and *B*). Tested in the *in vitro* transcription assays, a high concentration of the NTD did not block TFIIF but demonstrated a very slight inhibition of TTF2 activity (Fig. 2*C*), indicating that the NTD requires support from other domains of Gdown1 to have sufficient activity. The CTD has been predicted to have structural similarity to part of the RAP30 subunit of TFIIF (17), and we hypothesized that the CTD might compete with TFIIF in binding Pol II. Full-length Gdown1 and Gdown1 CTD were titrated onto EECs before adding TFIIF or TTF2 and performing a 7-min chase. Full-length Gdown1 completely blocked TFIIF at 0.02 pmol/reaction, whereas the Gdown1 CTD achieved the same blocking activity at 20 pmol (Fig. 2*D*). However, the CTD alone showed very slight inhibitory effect on TTF2 (Fig. 2*E*). Together, these results strongly suggest that the CTD of Gdown1 is primarily responsible for inhibiting TFIIF, although the full-length protein shows a much stronger block probably because of its increased ability to bind to Pol II.

##### A Phosphorylated Form of Gdown1 Has Altered Activity

**FIGURE 3.**
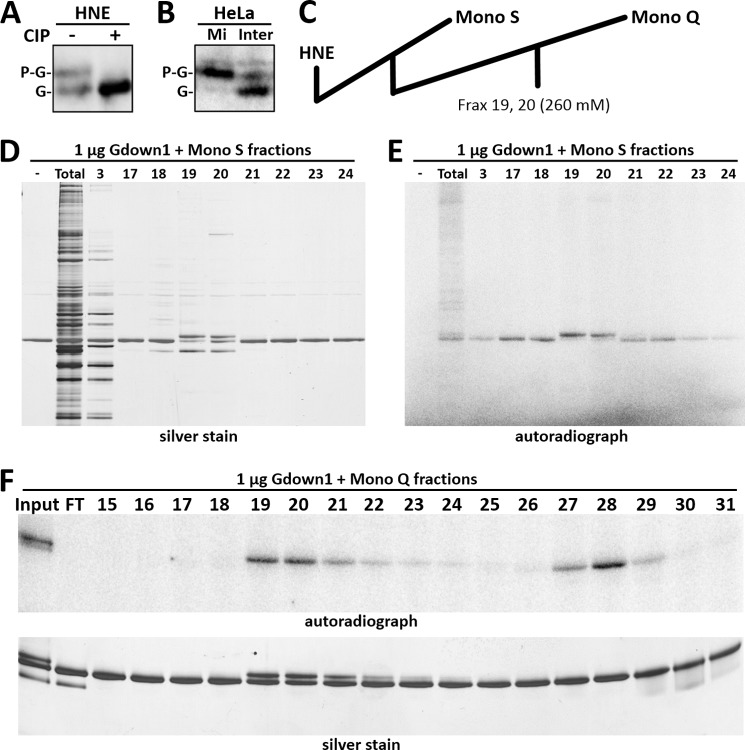
**Phosphorylation of Gdown1 in cells and *in vitro*.** *A*, Western blot of HNE without or after treatment with calf alkaline phosphatase (*CIP*), using antibodies to Gdown1. Bands corresponding to Gdown1 (*G*) and phosphorylated Gdown1 (*P-G*) are indicated. *B*, Western blot of mitotic (*Mi*) and interphase (*Inter*) HeLa cells with Gdown1 (*G*) and phosphorylated Gdown1 (*P-G*) indicated. *C*, diagram of HNE fractionation. Fractions eluting at approximately 280 mm KCl on Mono S column were further fractionated on Mono Q. Kinase assays were performed as described under “Experimental Procedures” using 1 μg of Gdown1 and 4 μl of the indicated fractions. Reactions were then analyzed by SDS-PAGE followed by silver staining and phosphorimaging. *D*, Mono S, silver stain. *E*, Mono S, phosphorimage. *F*, Mono Q, phosphorimage (*upper panel*) and silver stain (*lower panel*).

Western blotting using affinity-purified Gdown1 antibodies consistently detected two bands at approximately 50 kDa from HeLa whole cell extract (16). The two bands were also observed in HNE (Fig. 3*A*). Treating HNE with calf alkaline phosphatase caused the disappearance of the upper band and increased intensity of the lower (Fig. 3*A*). This suggests that the upper band is a phosphorylated form of Gdown1 with reduced mobility in SDS-PAGE. Phosphorylation of Gdown1 on multiple residues has been reported previously, but no functional consequences were described (17, 27). Given that Gdown1 blocks TTF2 function and TTF2 causes global Pol II termination during mitosis (19), we wondered whether Gdown1 might be phosphorylated during mitosis. Mitotic cells were shaken off and collected from a population of adherent HeLa cells, whereas the remaining cells were harvested as interphase cells. A Western blot of whole cell extracts indicated that the predominant form of Gdown1 in mitotic cells is the shifted, phosphorylated form (Fig. 3*B*). This suggests that mitotic phosphorylation of Gdown1 might allow termination by TTF2.

To characterize the phosphorylated Gdown1 biochemically, we sought to obtain a phosphorylated form with minimal contamination of the unmodified form. In initial kinase assays in which high levels of recombinant Gdown1 were incubated with HNE, a fraction of the Gdown1 exhibited the shifted mobility (data not shown). HNE was fractionated on Mono S followed by Mono Q (Fig. 3*C*), and each fraction was tested for Gdown1 kinase activity. On Mono S most fractions caused incorporation of label from [γ-^32^P]ATP, but two fractions also led to the shift (Fig. 3, *D* and *E*). These two fractions were further fractionated on Mono Q, and a shifting kinase was separated from a nonshifting kinase (Fig. 3*F*).

**FIGURE 4.**
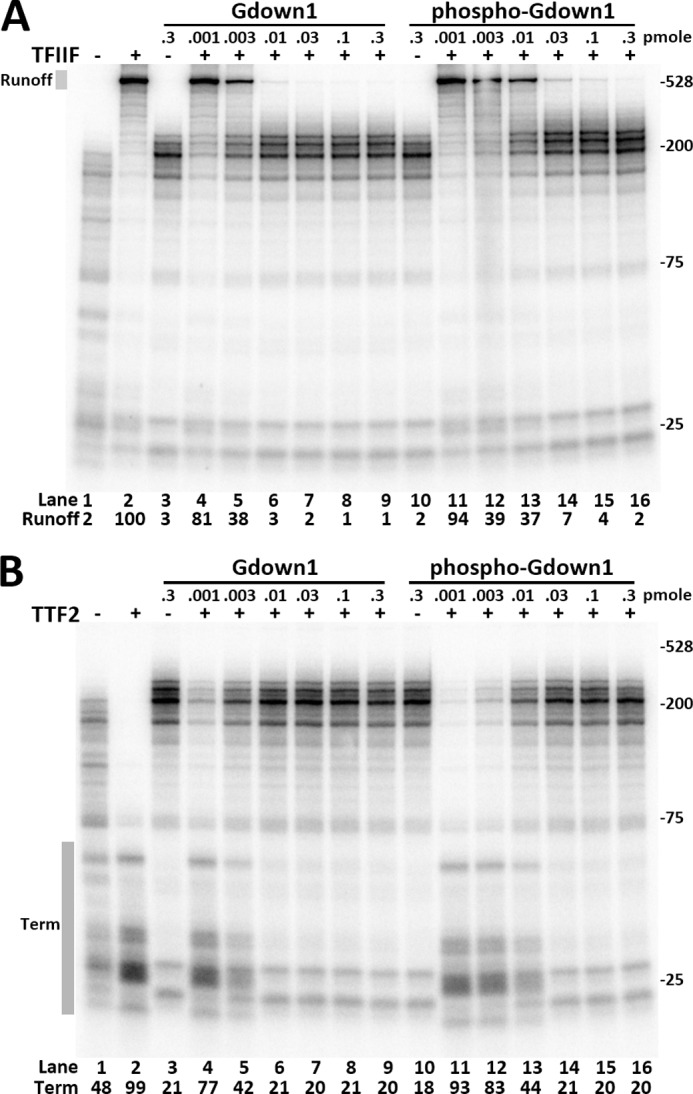
**Phosphorylated Gdown1 has reduced activity in blocking TFIIF and TTF2.** *A*, indicated amounts of Gdown1 or phosphorylated Gdown1 (*phospho-Gdown1*) were incubated with EECs before adding 0.1 pmol of TFIIF as indicated and chased for 7 min. Intensity of the runoff region in each *lane* was quantified and normalized against the TFIIF only lane. *B*, identical reactions to *A* except using 0.04 pmol of TTF2/reaction instead of TFIIF. The termination region (*Term*) in each *lane* was quantified and is shown as its percentage of the total lane intensity.

To examine the effect of the shifting phosphorylation on the function of Gdown1, phospho-Gdown1 was generated using Mono Q fraction 19 in a kinase assay with only nonradioactive ATP. The kinase reaction was stopped with EDTA, and the phosphorylation of Gdown1 was >90% complete as judged by silver staining after SDS-PAGE. Increasing levels of the unmodified Gdown1 in elongation assays caused the expected inhibition of both TFIIF and TTF2 (Fig. 4). At 0.003 pmol of Gdown1 the runoff signal caused by TFIIF was reduced to 38% (Fig. 4*A*, *lane 5*), and the termination signal caused by TTF2 was reduced to 42% (Fig. 4*B*, *lane 5*). A 3.3-fold increase in Gdown1 saturated the Pol II leading to almost complete inhibition of TFIIF and TTF2. The effects seen when phospho-Gdown1 was titrated onto EECs were similar except that a little more than 3 times phospho-Gdown1 was required to achieve the same level of inhibition compared with unphosphorylated Gdown1 (Fig. 4, *A* and *B*, *lanes 13*). These results demonstrate that the phosphorylation weakens the effect of Gdown1 in blocking TFIIF and TTF2. Blocking at higher concentrations could be explained by unphosphorylated Gdown1 (present because of incomplete phosphorylation) becoming dominant because of essentially irreversible binding or by an increased fractional occupancy of the elongation complex with a weaker binding phosphorylated protein.

**FIGURE 5.**
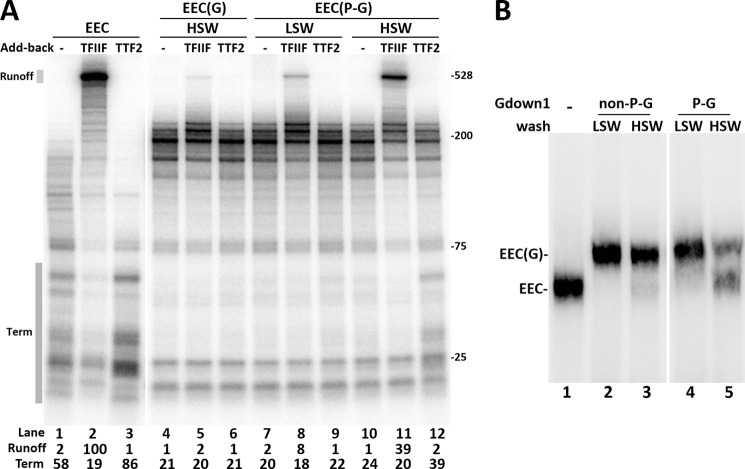
**Phosphorylated Gdown1 has reduced affinity for Pol II.** *A*, Gdown1 or phosphorylated Gdown1 (*P-G*) was incubated with EECs and then washed by LSW or HSW as indicated. TFIIF or TTF2 were added back as indicated before a 6-min chase. Intensity of the runoff region in each *lane* was quantified and normalized against the TFIIF only lane. The termination region (*Term*) in each *lane* was quantified and is shown as its percentage of the total lane intensity. *B*, EC-EMSA. Isolated EECs were incubated with 1 pmol of the nonphosphorylated Gdown1 (*non-P-G*) or Ser-270-phosphorylated Gdown1 (*P-G*) before being washed with 60 mm KCl (LSW) or 1.6 m KCl (HSW) as indicated. The complexes were digested off the beads by SacI at 37 °C for 15 min and run on a 4% native acrylamide gel for 2.5 h at 6 W before phosphorimaging.

We then tested whether the reduced effects of phospho-Gdown1 were because of a reduced affinity for Pol II. EECs were incubated with saturating levels of either unmodified Gdown1 or the phospho-Gdown1 for 10 min at room temperature before washing with LSW (60 mm KCl) or HSW (1.6 m KCl). The complexes were then incubated with TFIIF or TTF2 before being chased. After a HSW of EEC(G) the elongation complexes remained largely resistant to TFIIF and TTF2 (Fig. 5*A*, *lanes 4–6*) (16). After LSWs, EEC with phospho-Gdown1 (EEC(P-G)) showed a slightly increased response to TFIIF and TTF2 (Fig. 5*A*, compare *lanes 4–6* with *7–9*), although the increased response to TTF2 might be too small to be considered significant. This indicates that phospho-Gdown1 is still able to bind to Pol II, but may have a slightly weakened ability to do so. This became very apparent after high salt wash. EEC(P-G) showed significant responses to TFIIF and TTF2, suggesting that phosphorylation of Gdown1 reduces its affinity for Pol II (Fig. 5, *lanes 11–12*). To confirm this, EC-EMSA was performed, and it demonstrated that a small fraction of phospho-Gdown1 was removed from EECs after low salt wash and that a significant fraction of it was removed from the complexes by high salt wash (Fig. 5*B*). We conclude that phosphorylation of Gdown1 weakens its affinity for Pol II and this allows partial function of TTF2 and TFIIF.

##### Phosphorylation of Gdown1 on Ser-270 Reduces Its Affinity for Pol II

**FIGURE 6.**
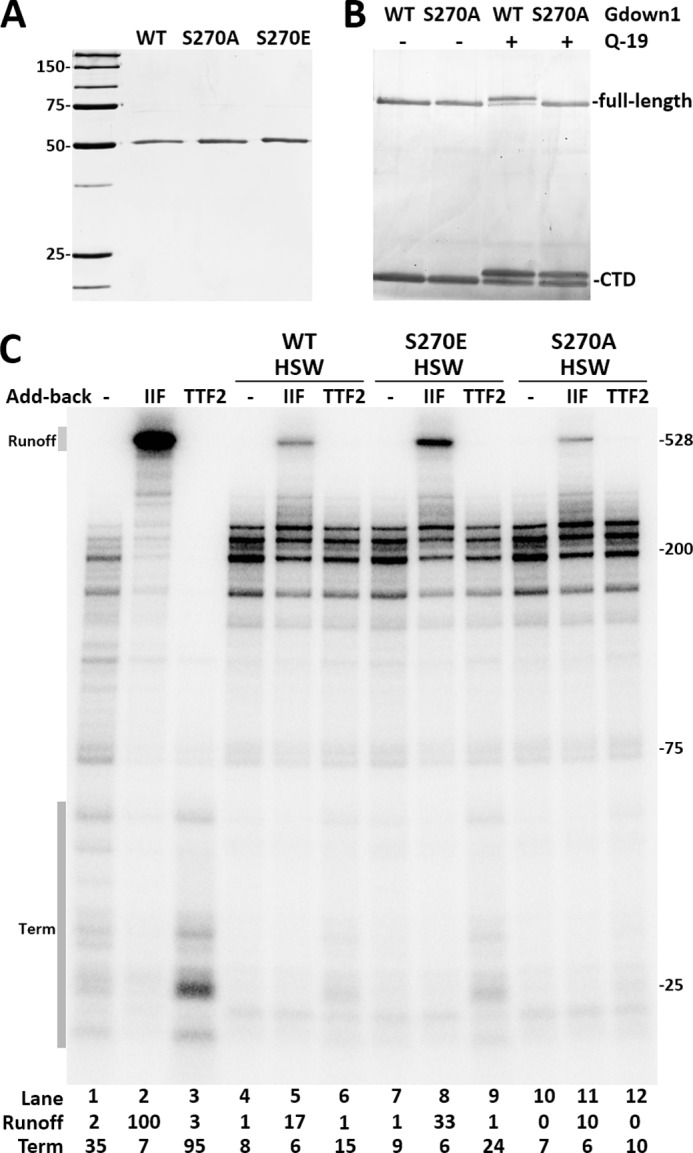
**Identification and confirmation of the site of phosphorylation.** *A*, purified wild-type (WT) and mutant Gdown1 proteins were analyzed by SDS-PAGE and silver staining. *B*, wild-type and mutant full-length Gdown1 proteins were mixed with the wild-type Gdown1-CTD protein and subjected to mock kinase reactions (−) or reactions containing Mono Q fraction 19 (+). *C*, wild-type or mutant Gdown1 proteins were incubated with EECs and then washed with high salt (HSW). TFIIF or TTF2 were added back as indicated before a 7-min chase. Intensity of the runoff region in each *lane* was quantified and normalized against *lane 2*. The termination region (*Term*) in each *lane* was quantified and is shown as its percentage of the total lane intensity.

Gdown1 phosphorylated by the kinase fraction was affinity purified by nickel-nitrilotriacetic acid and subjected to mass spectrometric analyses to identify the phosphorylation site. Only one phosphopeptide was identified, and it contained phosphorylated serine 270. Gdown1 mutations changing serine 270 to alanine (S270A) and glutamic acid (S270E) were produced to block or mimic phosphorylation of Ser-270, respectively. By SDS-PAGE, the S270A mutant showed mobility identical to the wild-type whereas the S270E mutant ran slightly slower (Fig. 6*A*). To determine that Ser-270 was indeed the site of phosphorylation, both wild-type and the S270A mutant were individually mixed with a wild-type CTD protein that contains Ser-270 and subjected to a kinase reaction without or with the kinase fraction. Both wild-type full-length Gdown1 and the CTD were shifted by the phosphorylation (Fig. 6*B*). Under identical conditions the S270A mutant did not shift although the wild-type CTD in the same reaction did (Fig. 6*B*). These results demonstrate that phosphorylation of Ser-270 is responsible for the mobility change.

To establish the functional significance of this Ser-270 phosphorylation, wild-type Gdown1 and the S270E and S270A mutants were incubated with EECs prior to extensive high salt washing. After the wash, Pol II with wild-type Gdown1 remained mostly resistant to TFIIF and TTF2 (Fig. 6*C*, *lanes 5* and *6*). The S270E mutant showed reduced resistance to HSW as significantly higher amount of the elongation complexes were stimulated by TFIIF, and an increased amount of termination was caused by TTF2 (Fig. 6*C*, *lanes 8* and *9*). However, the S270A mutant showed a slightly increased resistance to HSW compared with the wild-type. The Gdown1 CTD truncation that bears Ser-270 was fully phosphorylated by the Mono Q fraction 19 and tested for its ability to inhibit TFIIF as shown in Fig. 2*D*. Wild-type CTD was able to significantly inhibit TFIIF, but after phosphorylation no inhibition was detected (data not shown). The results with mutant proteins confirm Ser-270 as the site of phosphorylation and that phosphorylation of Ser-270 on Gdown1 leads to reduced inhibition of TFIIF and TTF2 through reduction in the affinity for Pol II.

## DISCUSSION

**FIGURE 7.**
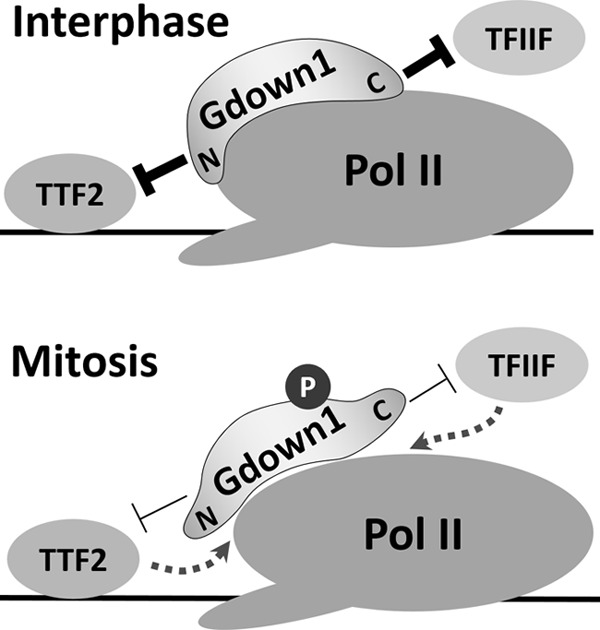
**Functional domains and regulation of Gdown1 by phosphorylation.** Gdown1 blocks TTF2 with the NTD and blocks TFIIF with the CTD. *A*, during interphase unmodified Gdown1 binds to Pol II tightly and strongly inhibits both TFIIF and TTF2. *B*, during mitosis Gdown1 is phosphorylated on Ser-270, and this reduces the affinity of Gdown1 for Pol II and allows TFIIF and TTF2 to function on Pol II.

Gdown1 was previously shown to have the dual function of blocking the action of TFIIF and TTF2 on Pol II, and here we have dissected functional domains of Gdown1 that are responsible for inhibiting the two factors (Fig. 7). We suggest that the NTD is primarily responsible for blocking the interaction of TTF2 with the polymerase. This is based on (i) conservation of LPDKG in both the NTD and in TTF2 and (ii) the preferential loss of inhibition of TTF2 in the LPDKG mutant. We suggest that the CTD blocks the interaction of TFIIF based on the ability of the CTD alone to block TFIIF, as well as the previously suggested sequence similarity between the RAP30 subunit of TFIIF and a region in the CTD (17). We do not suggest that the two domains act completely alone. In fact, our results indicate that both domains contribute significantly to the interaction of Gdown1 with Pol II. We have also determined that phosphorylation of Ser-270 results in reduced affinity to Pol II and increased activity of TFIIF and TTF2 on elongation complexes, and this reveals a potential mechanism for regulation of Gdown1 (Fig. 7).

During mitosis TTF2 causes global termination of Pol II (19, 28). Promoter-proximal paused polymerases including those modified by Gdown1 would be present during the beginning of mitosis and, in fact, might be the predominant form of Pol II engaged in transcription because of the increased resistance to TTF2 provided by Gdown1 (16). Our findings here provide a convenient method to release the effect of Gdown1 through Ser-270 phosphorylation. Ser-270 is followed by a proline, which suggests that the site could be a target of the cyclin-dependent kinases that are involved in regulating the entry and exit from mitosis (29). Such phosphorylation by a mitotic kinase would allow termination by TTF2, and the subsequent dephosphorylation of Gdown1 could allow it to recycle to its active form (Fig. 7).

The majority of Gdown1 ChIP-Seq signals accumulate around transcription start sites (16, 17), where initiation factors and Mediator are found (30–33). Gdown1 can outcompete the well characterized initiation factor TFIIF (34, 35) to inhibit formation of preinitiation complexes, and this inhibition could be overcome by Mediator (15, 17). EM studies have demonstrated that TFIIF can stabilize the Mediator-Pol II interaction (36), and TFIIF and Gdown1 interact with similar sites on Pol II (18); but because Mediator has been found in varying compositions (37, 38) and can respond to different activators (39) Gdown1 could play a role in regulating Mediator-Pol II interactions in the preinitiation complex. Gdown1 phosphorylation could provide a means to switch from Mediator-Pol II-Gdown1 to Mediator-Pol II-TFIIF. To further elucidate the regulation of Gdown1 during initiation, the use of a more defined transcription system will be required. In the early stages of elongation TTF2 has been shown to cause premature termination at positions close to transcription start sites (40). Phosphorylation at Ser-270 provides a feasible explanation for regulating premature termination.

An outstanding question is when Gdown1 phosphorylation at Ser-270 occurs. Does phosphorylation take place before or after Gdown1 binding to Pol II? This also adds to the existing question of when Gdown1 is incorporated into the transcription machinery. It would be logical to propose that Gdown1 can be phosphorylated during transcription because this would allow Pol II(G) elongation complexes to be terminated during mitosis. In the *in vitro* transcription assays described here, the phosphorylation at Ser-270 appears to only weaken, but not completely abolish, the Gdown1 affinity to elongating Pol II. It is possible that this modification is sufficient for TTF2 to terminate given a longer time or with the assistance of other factors. Also, other uncharacterized modifications or factors could also assist Gdown1 release.

Future work would include pursuing the identification of the kinase and a potential phosphatase. That information would indicate whether the regulation of Ser-270 phosphorylation is related primarily to the cell cycle or more broadly to transcription at all stages. The mutations of Gdown1 described here should be useful in determining how Gdown1 affects transcription in the context of other transcription factors.

## Footnotes

2 The abbreviations used are:

Pol II: RNA polymerase II
CTD: C-terminal domain
DRB: 5,6-dichloro-1-β-d-ribofuranosylbenzimidazole
DSIF: DRB sensitivity-inducing factor
EEC: early elongation complex
EEC(G): early elongation complexes with Gdown1
EEC(P-G): early elongation complexes with phospho-Gdown1
HNE: HeLa nuclear extract
HSW: high salt wash
LSW: low salt wash
NELF: negative elongation factor
NTD: N-terminal domain
Pol II(G): Pol II with Gdown1
P-TEFb: positive transcription elongation factor b
TFIIF: transcription factor IIF
TTF2: transcription termination factor 2.
